# Long-Term Survival in HIV Positive Patients with up to 15 Years of Antiretroviral Therapy

**DOI:** 10.1371/journal.pone.0048839

**Published:** 2012-11-07

**Authors:** Hamish McManus, Catherine C. O'Connor, Mark Boyd, Jennifer Broom, Darren Russell, Kerrie Watson, Norman Roth, Phillip J. Read, Kathy Petoumenos, Matthew G. Law, Australian HIV Observational Database

**Affiliations:** 1 The Kirby Institute, UNSW, Sydney, New South Wales, Australia; 2 RPA Sexual Health, Royal Prince Alfred Hospital, Sydney, New South Wales, Australia; 3 South Western Clinical School, UNSW, Sydney, New South Wales, Australia; 4 Central Clinical School, Sydney University, Sydney, New South Wales, Australia; 5 Department of Infectious Diseases, Nambour General Hospital, Nambour, Queensland, Australia; 6 Cairns Sexual Health Service, Cairns, Queensland, Australia; 7 The Alfred Hospital, Melbourne, Victoria, Australia; 8 Prahran Market Clinic, Prahran, Victoria, Australia; 9 Sydney Sexual Health Centre, Sydney, New South Wales, Australia; National Institute of Allergy and Infectious Diseases, United States of America

## Abstract

**Background:**

Life expectancy has increased for newly diagnosed HIV patients since the inception of combination antiretroviral treatment (cART), but there remains a need to better understand the characteristics of long-term survival in HIV-positive patients. We examined long-term survival in HIV-positive patients receiving cART in the Australian HIV Observational Database (AHOD), to describe changes in mortality compared to the general population and to develop longer-term survival models.

**Methods:**

Data were examined from 2,675 HIV-positive participants in AHOD who started cART. Standardised mortality ratios (SMR) were calculated by age, sex and calendar year across prognostic characteristics using Australian Bureau of Statistics national data as reference. SMRs were examined by years of duration of cART by CD4 and similarly by viral load. Survival was analysed using Cox-proportional hazards and parametric survival models.

**Results:**

The overall SMR for all-cause mortality was 3.5 (95% CI: 3.0–4.0). SMRs by CD4 count were 8.6 (95% CI: 7.2–10.2) for CD4<350 cells/µl; 2.1 (95% CI: 1.5–2.9) for CD4 = 350–499 cells/µl; and 1.5 (95% CI: 1.1–2.0) for CD4≥500 cells/µl. SMRs for patients with CD4 counts <350 cells/µL were much higher than for patients with higher CD4 counts across all durations of cART. SMRs for patients with viral loads greater than 400 copies/ml were much higher across all durations of cART. Multivariate models demonstrated improved survival associated with increased recent CD4, reduced recent viral load, younger patients, absence of HBVsAg-positive ever, year of HIV diagnosis and incidence of ADI. Parametric models showed a fairly constant mortality risk by year of cART up to 15 years of treatment.

**Conclusion:**

Observed mortality remained fairly constant by duration of cART and was modelled accurately by accepted prognostic factors. These rates did not vary much by duration of treatment. Changes in mortality with age were similar to those in the Australian general population.

## Introduction

Mortality has decreased for newly diagnosed HIV-positive patients since the inception of combination antiretroviral therapy (cART) [1], [2] and HIV infection can now be characterised as a manageable chronic condition. The nature of long-term survival with HIV is increasingly being revealed through the study of populations of patients with extended durations of exposure [3], [4]. Treatment can be complex with chronic pathologies associated with immunodeficiency, chronic viral infection and sociobehavioural factors. The accurate description of survival in HIV-positive populations today is therefore increasingly important in HIV management.

Long-term survival in HIV-positive populations with access to effective treatment appears to be approaching that of the general population [5]. Studies have shown declining rates of AIDS related death compared to non-AIDS related death since the introduction of cART [6], [7] and describe a need for increasing focus on chronic disease management and health promotion [8], [9]. All-cause mortality in patients who have achieved high CD4 cell count levels approaches that of the general population over time [10], although there is strong evidence that CD4 cell counts trend towards different plateaus according to pre cART levels [11], [12]. This suggests that long-term mortality can be associated with early uncontrolled viral replication and immune activation, and has led to contention about threshold levels of CD4 cell counts for treatment initiation.

Further, there is strong evidence associating immunologic resilience with age, and age at cART initiation has been associated with the rate and extent of immunologic recovery [13]. The effects of ageing on survival therefore need to be considered in addition to just the effects of increased duration of illness. However, studies of overall life expectancy in HIV-positive populations are often limited by insufficient data in older age groups [4] where rapid increases in general population mortality are observed. There remains a need to better understand long-term survival in ageing HIV-positive patients after prolonged cART.

The primary objective of this analysis is to measure all-cause mortality in adult HIV-positive patients receiving cART in Australia. Specifically we want to compare mortality rates in these patients with those of the general population, over the long-term, and examine how these rates are affected by duration of treatment when adjusted for prognostic factors. A secondary objective of this analysis is to examine the effects of ageing on mortality in HIV-positive populations relative to the general population.

## Methods

### Study population

The Australian HIV Observational Database (AHOD) is an observational clinical cohort study of patients with HIV infection seen at 27 clinical sites throughout Australia. AHOD utilises methodology which has been described in detail elsewhere [14]. Briefly, data are transferred electronically to the Kirby Institute at the University of New South Wales every 6 months. Core data variables include: sex; date of birth; date of most recent visit; HIV exposure; hepatitis B virus (HBV) surface antigen status; hepatitis C virus (HCV) antibody status; CD4 and CD8 counts; HIV viral load; antiretroviral treatment data; AIDS-defining illnesses; and date and cause of death. Prospective data collection commenced in 1999, with retrospective data provided where available.

Ethics approval for the study was granted by the University of New South Wales Human Research Ethics Committee, and all other relevant institutional review boards. Written informed consent was obtained from participating individuals. All study procedures were developed in accordance with the revised 1975 Helsinki Declaration.

This analysis included all patients who had been recruited to AHOD prior to 31 March 2011 and who had at least 1 subsequent clinical visit after the date of first recorded cART (defined as the use of 3 or more antiretrovirals from more than 1 class).

The study endpoint was mortality. The method of collection of mortality in AHOD has been described in detail elsewhere [15]. Briefly, AHOD uses standardised cause of death (CoDe) forms based on versions of the Data Collection on Adverse Events of Anti-HIV Drugs (D∶A∶D) cohort study [16] (www.cphiv.dk/CoDe). These are reviewed by an independent HIV specialist clinician at the Kirby Institute who determines the primary and secondary causes of death or recommends additional information be provided by the study site as required.

The covariates considered were: sex; age; mode of HIV exposure (men who have sex with men (MSM), heterosexual, injecting drug user (IDU), other/unknown); time updated instance of first AIDS defining illness (ADI); mono or dual antiretroviral treatment prior to first cART; HCV antibody (no/not tested, ever positive); HBV surface antigen (HBVsAg) (no/not tested, ever positive); time updated CD4 cell count (closest prior or up to 30 days after −<350, 350–499, ≥500 cells/µl); time updated viral load (closest prior or up to 30 days after −≤400, >400 copies/ml); year of first cART (≤1995, 1996–99, 2000–2003, ≥2004); and time updated number of regimens received (a new regimen was defined as the commencement of 1 or more new antiretroviral drugs and for more than 14 days).

### Statistical Analysis

Follow up was calculated from the start of cART and censored at the earlier of death, lost to follow up or 31 March 2011 (cohort censoring date). Lost to follow up date was defined as the most recent clinic visit if no clinic visit had been recorded in the 12 months prior to 31 March 2011. We used an intent-to-continue treatment approach and ignored any changes to treatment after baseline including periods off treatment. This approach avoids problems of modelling causal effects of patients stopping cART.

Standardised mortality ratios (SMR) across prognostic characteristics were calculated referent to the Australian Bureau of Statistics (ABS) general population mortality rates by age group, gender and calendar year [17], [18]. SMRs were also calculated by years of cART for given levels of time updated CD4 cell count and for given levels of time updated viral load.

Cox proportional hazards models were initially developed to identify important survival covariates. Initially univariate models were developed and all significant univariate predictors were considered for inclusion in multivariate models. Final multivariate models were developed using a backwards stepwise approach to reduce to a parsimonious set of statistically significant (2p<0.05) covariates. A sensitivity analysis was conducted using Cox proportional hazards models of survival in patients who had commenced cART after 1 January 1999.

Parametric survival-time Weibull models using clustered variance estimators were developed based on these covariates, and used to investigate survival trends by age. Probability of 10 year survival was calculated for a range of representative prognostic covariate values. Results were compared with 10 year survival rates for general population Australian males 2007–2009 using ABS data [19].

Data were analysed using Stata version 12 (Stata Corporation, College Station, Texas, USA).

## Results

### Patient characteristics

Of 3,173 people in AHOD, 2,675 had commenced cART and had at least 1 subsequent clinical visit or result recorded post therapy. During 15,936 patient years of follow-up 206 deaths were observed. The rate of loss to follow-up of patients was 40.4 per 1000 person years of follow-up (95% CI: 37.3–43.6). The number of patients at first cART by selected characteristics is shown in Table 1. At first cART the population was predominantly male (94%), aged from 30–49 (70%) and the main exposure category was MSM (75%). The median CD4 cell count at cART commencement was 282 cells/µL (interquartile range (IQR): 151–427) and viral load was 58,942 copies/ml (IQR: 1,000–199,263). The majority of patients (60%) had no prior treatment at cART commencement.

**Table 1:** Patient characteristics at cART commencement.

	N = 2,675	(%)
**Sex**		
Female	149	(6)
Male	2,526	(94)
**Age at first cART (years)**		
Median (IQR)	42 (36–49)	
<30	364	(14)
30–39	1074	(40)
40–49	791	(30)
50–59	354	(13)
60–69	81	(3)
70–79	11	(0)
**Mode of HIV exposure**		
MSM	2,001	(75)
IDU	154	(6)
HET	248	(9)
Other	272	(10)
**ADI**		
No	2,271	(85)
Yes	404	(15)
**HCV (ever)**		
No/not tested	2390	(89)
Yes	285	(11)
**HBVsAg (ever)**		
No/not tested	2549	(95)
Yes	126	(5)
**CD4 (cells/µL)**		
Median (IQR)	282 (151–427)	
<350	1,316	(49)
351–499	408	(15)
≥500	379	(14)
Missing	572	(21)
**Viral Load (copies/ml)**		
Median (IQR)	58,942 (10,000–199,263)	
≤400	166	(6)
>400	1,722	(64)
Missing	787	(29)
**Year of first cART**		
≤1995	138	(5)
1996–1999	1593	(60)
2000–2003	390	(15)
≥2004	554	(21)
**Regimen number at first cART**		
1^st^	1,600	(60)
2^nd^ or 3^rd^	824	(31)
4^th^ or more	251	(9)

### Mortality rates

Observed mortality by patient characteristics is shown in Table 2. The overall mortality rate was 12.9 deaths/1000 patient years (95% CI: 11.3–14.8) and the overall SMR was 3.5 (95% CI: 3.0–4.0). An increase was observed in mortality rates with increasing age from 10.8 deaths/1000 patient years (95% CI: 4.0–28.7) in patients aged <30, to 21.6 deaths/1000 patient years (95% CI: 15.6–30.0) in patients aged ≥60. A decrease towards general population level was observed in SMRs by increasing age from patients aged <30 (SMR 12.4; 95% CI 4.7–33.0) to patients aged ≥60 (SMR 1.4; 95% CI 1.0–2.0). An increased SMR was observed for patients with mode of HIV exposure through IDU (SMR 11.3; 95% CI 7.3–17.5). SMRs were increased for patients with recorded HCV ever (SMR 7.2; 95% CI 5.1–10.0) compared to those without HCV ever (SMR 3.1; 95% CI 2.7–3.7), and similarly for patients with recorded HBVsAg ever (SMR 10.6; 95% CI 7.0–15.9) compared to those without HBVsAg ever (SMR 3.2; 95% CI 2.8–3.7).

**Table 2:** Mortality by patient characteristics.

	Deaths	Expected	PYs (000's)	Mortality rate(95% CI)	SMR (95% CI)
**All mortality**					
	206	59.4	15.9	12.9 (11.3, 14.8)	3.5 (3.0, 4.0)
**Sex**					
Female	8	1.3	0.9	9.3 (4.6, 18.5)	6.0 (3.0, 11.9)
Male	198	58	15.1	13.1 (11.4, 15.1)	3.4 (3.0, 3.9)
**Age**					
0–29	4	0.3	0.4	10.8 (4.0, 28.7)	12.4 (4.7, 33.0)
30–39	38	4.4	3.8	9.9 (7.2, 13.6)	8.6 (6.2, 11.8)
40–49	73	12.4	6.2	11.8 (9.4, 14.8)	5.9 (4.7, 7.4)
50–59	55	17.2	3.9	14.2 (10.9, 18.5)	3.2 (2.5, 4.2)
60–90	36	25.1	1.7	21.6 (15.6, 30.0)	1.4 (1.0, 2.0)
**Mode of HIV exposure**					
MSM	153	46.4	12.2	12.6 (10.7, 14.7)	3.3 (2.8, 3.9)
IDU	20	1.8	0.9	22.5 (14.5, 34.9)	11.3 (7.3, 17.5)
HET	12	4.9	1.4	8.8 (5.0, 15.5)	2.5 (1.4, 4.4)
OTHER	21	6.4	1.5	13.8 (9.0, 21.1)	3.3 (2.1, 5.0)
**ADI prior to cART**					
No	155	48.5	13.3	11.6 (9.9, 13.6)	3.2 (2.7, 3.7)
Yes	51	10.9	2.6	19.5 (14.8, 25.6)	4.7 (3.6, 6.2)
**HCV (ever)**					
No/no tested	172	54.6	14.2	12.2 (10.5, 14.1)	3.1 (2.7, 3.7)
Yes	34	4.8	1.8	19.1 (13.6, 26.7)	7.2 (5.1, 10.0)
**HBVsAg (ever)**					
No/not tested	183	57.2	15.2	12.1 (10.4, 13.9)	3.2 (2.8, 3.7)
Yes	23	2.2	0.8	30.0 (20.0, 45.2)	10.6 (7.0, 15.9)
**CD4 (cells/µl)** 1					
<350	131	15.3	4.1	32.0 (27.0, 38.0)	8.6 (7.2, 10.2)
350–499	32	15.6	4.1	7.7 (5.5, 11.0)	2.1 (1.5, 2.9)
≥500	43	28.5	7.7	5.6 (4.1, 7.5)	1.5 (1.1, 2.0)
**Viral Load (copies/ml)** 1					
≤400	99	47.5	11.8	8.4 (6.9, 10.2)	2.1 (1.7, 2.5)
>400	107	11.9	4.2	25.8 (21.3, 31.1)	9.0 (7.5, 10.9)
**Treatment prior to cART**					
No	81	29.4	8.4	9.7 (7.8, 12)	2.8 (2.2, 3.4)
Yes	125	30	7.6	16.5 (13.9, 19.7)	4.2 (3.5, 5.0)
**Year of cART commencement**					
≤1995	17	4.3	1.1	16.1 (10.0, 25.9)	3.9 (2.4, 6.3)
1996–1999	159	44.7	11.7	13.6 (11.6, 15.9)	3.6 (3.0, 4.2)
2000–2003	24	6.8	2.1	11.7 (7.8, 17.5)	3.5 (2.4, 5.3)
≥2004	6	3.5	1.1	5.3 (2.4, 11.8)	1.7 (0.8, 3.8)
**Regimen number** 1					
1st	12	6.0	2.1	5.7 (3.2, 10.0)	2.0 (1.1, 3.5)
2nd or 3rd	29	16.6	4.7	6.1 (4.3, 8.8)	1.7 (1.2, 2.5)
4th or more	165	36.8	9.1	18.1 (15.6, 21.1)	4.5 (3.9, 5.2)

1 Time updated.

A large difference was observed in mortality rates for patients with CD4 cell counts of <350 cells/µl (Mortality rate (MR) 32.0; 95% CI 27.0–38.0) compared to patients with CD4 cell counts of 350–499 cells/µl (MR 7.7; 95% CI 5.5–11.0); and CD4 cell counts of ≥500 cells/µl (MR 5.6; 95% CI 4.1–7.5). Similarly the SMR for patients with CD4 cell counts of <350 cells/µl (SMR 8.6; 95% CI 7.2–10.2) was much higher than for patients with CD4 cell counts of 350–499 cells/µl (SMR 2.1; 95% CI 1.5–2.9); and for patients with CD4 cell counts of ≥500 cells/µl (SMR 1.5; 95% CI 1.1–2.0) which was slightly higher than population level mortality. An increased SMR was observed for patients with viral loads of >400 copies/ml (SMR 9.0; 95% CI 7.5–10.9) compared to patients with viral loads of ≤400 copies/ml (SMR 2.1; 95% CI 1.7–2.5).

**Figure 1:**
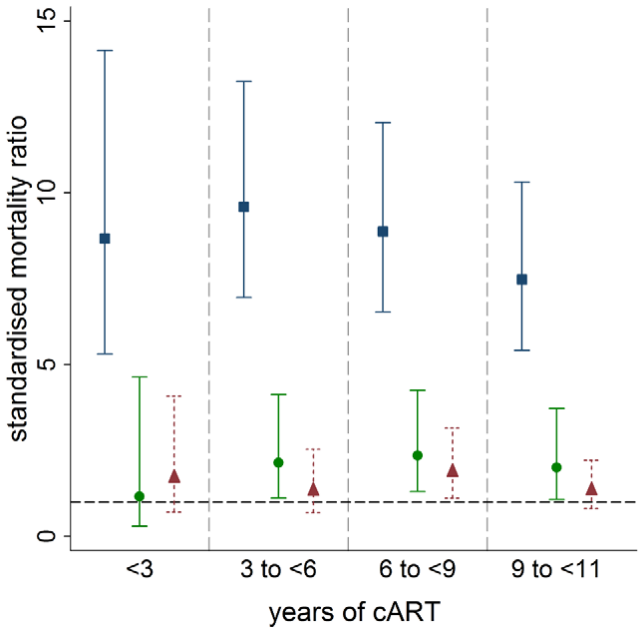
SMRs and 95% confidence intervals by years of cART and time updated CD4 cell count. Blue/square markers represent patients with CD4<350 cells/µl. Green/round markers represent patients with CD4 from 350 to 499 cells/µl. Red/triangular markers represent patients with CD4≥500 cells/µl. Grey/horizontal dashed line represents SMR of 1.

SMRs by time updated CD4 cell count category by years that a patient has been receiving cART are shown in Figure 1. SMRs for patients with CD4 cell counts <350 cells/µL were much higher than for patients with higher CD4 cell counts across all durations of cART. SMRs for patients with CD4 cell counts of 350–499 cells/µL were in the range 1.2 to 2.4. SMRs for patients with CD4 cell counts of ≥500 cells/µL were consistently closer to population level in the range 1.3 to 1.9.

**Figure 2:**
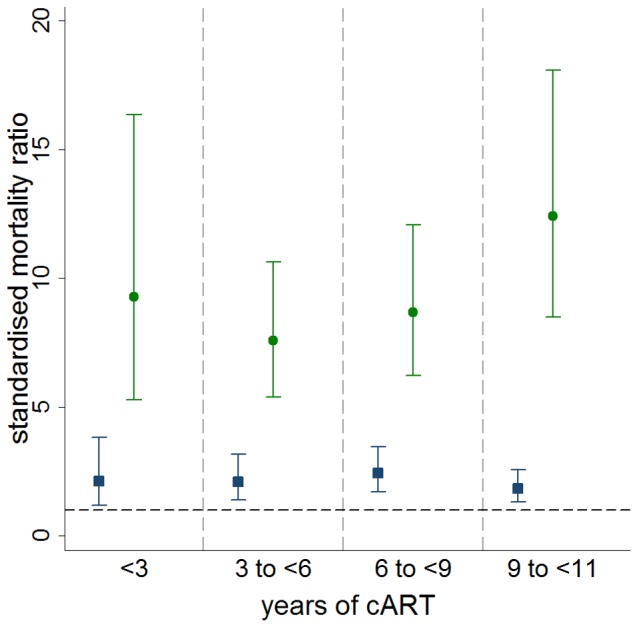
SMRs and 95% confidence intervals by years of cART and time updated HIV viral load. Blue/square markers represent patients with viral load ≤400 copies/ml. Green/round markers represent patients with HIV viral load >400 copies/ml. Grey/horizontal dashed line represents SMR of 1.

SMRs by time updated viral load category by years that a patient has been receiving cART are shown in Figure 2. SMRs for patients with viral loads of ≤400 copies/ml were relatively constant by years of cART (in the range 1.8 to 2.4). SMRs for patients with viral loads of >400 copies/ml were much higher (in the range 7.6 to 12.4) across all durations of cART and showed an upward trend after 3 years of cART.

### Prognostic model

Univariate and multivariate Weibull model predictors of survival are shown in Table 3. Univariate predictors associated with survival were time updated age (p = 0.003); mode of HIV exposure through IDU (p = 0.011); time updated instance of first ADI (p<0.001); HCV ever (p = 0.019); HBVsAg ever (p<0.001); time updated CD4 cell count (p<0.001); time updated viral load (p<0.001); treatment prior to cART (p<0.001); and time updated number of regimens received (p<0.001). Predictors retained in the multivariate model were time updated age (p<0.001); mode of HIV exposure through IDU (p = 0.041); time updated instance of first ADI (p<0.001); HBVsAg ever(p<0.001); time updated CD4 cell count (p<0.001); time updated viral load (p<0.001); and time updated number of regimens received (p<0.001).

**Table 3:** Weibull model predictors of survival for patients commencing cART^1^.

	Univariate			Multivariate^2^		
	Hazard (95% CI)	p	ρ^3^	Hazard (95% CI)	p	ρ^3^
**Sex**						
Female	ref					
Male	1.41 (0.69, 2.87)	0.343				
**Age** 4						
	1.02 (1.01, 1.04)	0.003		1.04 (1.02, 1.05)	<0.001	
**Mode of HIV exposure**						
Not IDU	ref			ref		
IDU	1.83 (1.15, 2.90)	0.011		1.63 (1.02, 2.60)	0.041	
**ADI** 4						
No	ref			ref		
Yes	2.44 (1.84, 3.23)	<0.001		1.62 (1.22, 2.14)	<0.001	
**HCV (ever)**						
No/not tested	ref					
Yes	1.56 (1.07, 2.25)	0.019				
**HBVsAg (ever)**						
No/not tested	ref			ref		
Yes	2.49 (1.61, 3.86)	<0.001		2.31 (1.51, 3.53)	<0.001	
**CD4(cells/µL)** 4						
<350	ref		<0.001	ref		<0.001
350–499	0.24 (0.16, 0.36)	<0.001		0.33 (0.23, 0.49)	<0.001	
≥500	0.17 (0.12, 0.24)	<0.001		0.29 (0.20, 0.40)	<0.001	
**Viral Load(count/ml)** 4						
≤400	ref			ref		
>400	3.17 (2.41, 4.18)	<0.001		2.57 (1.94, 3.41)	<0.001	
**Treatment prior to cART**						
No	ref					
Yes	1.67 (1.25, 2.23)	<0.001				
**Year of cART commencement**						
≤1995	ref		0.173			
1996–1999	0.85 (0.50, 1.42)	0.531				
2000–2003	0.73 (0.37, 1.45)	0.371				
≥2004	0.33 (0.12, 0.91)	0.032				
**Regimen number** 4						
1st	ref		<0.001	ref		<0.001
2nd or 3rd	1.26 (0.63, 2.54)	0.515		1.10 (0.54, 2.21)	0.800	
4th or more	4.19 (2.19, 8.02)	<0.001		2.66 (1.38, 5.13)	0.004	

1 2675 patients, 15936 years of follow up, 206 deaths.

2 Covariates selected by backwards stepwise selection from significant univariate predictors.

3 Wald test for homogeneity for categorical covariates.

4 Time updated.

**Figure 3:**
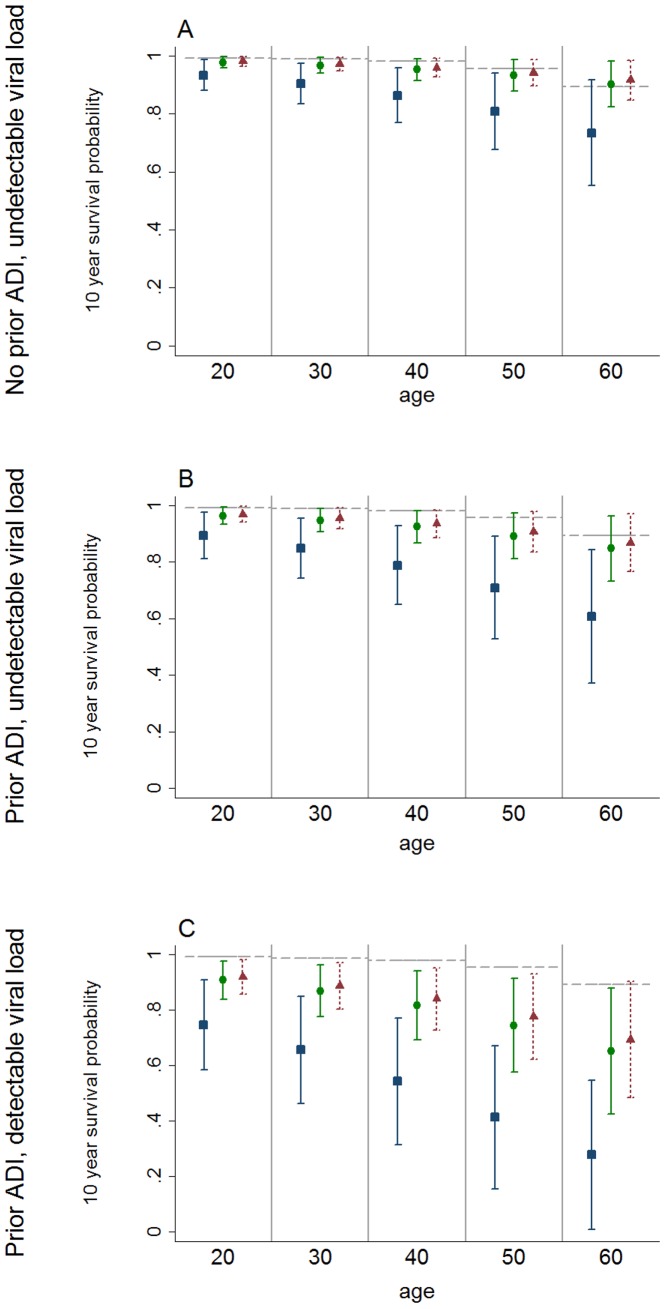
10 year survival probabilities and 95% confidence intervals by age and time updated CD4 count. Blue/square markers represent patients with CD4 less than 350 cells/µl. Green/round markers represent patients with CD4 from 350 to 499 cells/µl. Red/triangular markers represent patients with CD4≥500 cells/µl. Grey/horizontal dashed line represents Australian males 2008–09. These plots apply to patients on 4th or greater regimen, no prior HBVsAg, non-IDU mode of exposure and specified existing viral load and incidence of ADI. Plot (A) shows 10 year survival probabilities for patients with viral load ≤400 copies/ml and with no prior ADI. Plot (B) shows 10 year survival probabilities for patients with viral load ≤400 copies/ml and with prior ADI. Plot (C) shows 10 year survival probabilities for patients with viral load >400 copies/ml and with prior ADI.

Weibull model 10 year survival probabilities for specified ages by CD4 cell count are shown in Figure 3. For illustrative purposes we present 10 year survival probabilities of patients on their 4^th^ or greater regimen with no HBVsAg ever and mode of exposure other than IDU because these were the most representative observed states based on duration of analysis time. Scenarios are presented variously controlling for incidence of prior ADI and viral load. Survival probabilities for patients with CD4 cell counts of ≥350 cells/µL did not differ significantly from those of the general population for all age groups when controlling for other model covariates. Survival probabilities for patients maintaining CD4 cell counts of ≥500 cells/µL and controlling for other model covariates were similar to general population levels for all age groups: the 10 year survival probability for a 20 year old HIV-positive person controlling for other model covariates was 0.98 (95% CI 0.96–1.00) compared to 0.99 for 20 year olds in the male general population [19]; and for a 60 year old was 0.91 (95% CI 0.85–0.98) compared to 0.89 for 60 year olds in the male general population [19]. The decrease in survival probabilities associated with increase in age was less for patients with CD4 cell counts ≥350 cells/µL compared to patients with CD4 cell counts of <350 cells/µL. Prior ADI, increased viral load, and mode of HIV exposure through IDU were also associated with significantly reduced 10 year survival probabilities compared to the general population. Survival probabilities given the presence of 1 or more of these conditions decreased proportionally more with increasing age and relative to the general population compared to survival without these conditions.

In a sensitivity analysis, 10 year survival probabilities for a multivariate model of survival in patients who had commenced cART after 1 January 1999 were similar to those developed in the primary analysis (results not shown).

## Discussion

In this analysis from AHOD, overall mortality in adult HIV-positive patients receiving cART was found to be higher than general population level mortality, but mortality rates were close to general population levels in patients with high CD4 cell counts (especially above 500 cells/µL). There was no evidence of increased mortality by duration of cART when controlling for CD4 cell count level. There was no observed evidence of changes in mortality associated with age relative to the general population after controlling for time updated incidence of prior AIDS, mode of HIV exposure, time updated CD4 cell count, time updated viral load and time updated regimen number.

Prognostic indicators in our study are consistent with other studies [14], [20], [21]. In particular, our results confirm the importance of immunological reconstitution as a biomarker for long-term survival in HIV-positive populations and show the similarity between mortality in patients with CD4 cell counts above 350 cells/µL (350–499 cells/µL and ≥500cells/µL) and large increases in mortality below this level. Our results are very similar to those published recently in a study by the COHERE collaboration: SMR was 1.8 for CD4 cell count of 350–499 cells/µL and 1.5 for CD4 cell count of ≥500 cells/µL) [10]. We found that, at increased CD4 cell count levels, observed survival approaches that of the general population, although average rates at these levels remained slightly higher than those of the general population. This reflects the findings of recent studies [10], [22] and, given the association between CD4 cell count levels at commencement of cART and capacity for immunological recovery, reinforces the notion that CD4 cell count of 350 cells/µl is an important treatment threshold. Our results support the importance of ongoing immunological reconstitution as a treatment priority.

There was no observed association between duration of treatment and survival when adjusted for CD4 cell count level. At CD4 cell counts less than: 350 cells/µL there was a mild decrease in SMRs by duration of cART but rates remained much higher than population level; while above this level, average rates remained fairly constant, and just above population level. Although we observed a decrease in SMRs associated with recent cART commencement and year of cART commencement was a significant univariate predictor of survival, it was not a significant covariate in multivariate models. We also observed slightly decreased SMRs for patients with less than 3 years of cART experience compared to those with 3–6 years cART experience when stratified by CD4 cell count level, and these rates are weighted heavily by more recent cART starters. However these differences were not significant. These findings can be contrasted with a recent study by the Antiretroviral Therapy Cohort Collaboration of decreasing mortality by period of cART initiation (1996–99, 2000–02, 2003–05) [4]. In that study duration of treatment was associated with decreasing SMRs for each period of cART initiation, but these differences were not adjusted for CD4 cell count levels, and included earlier periods of follow-up than those used in this study.

This analysis provides a robust assessment of the association between age and survival for this population. Decreasing SMRs were observed with age, which adjust for different distributions of age, gender and temporal structure of AHOD compared to the general Australian population. However these ratios do not adjust for additional covariate factors, such as changes in CD4 cell count and viral load, because of limited sample size. Instead, survival models of the data demonstrated that values of, and decreases in 10 year survival probabilities over the range of ages included in these analyses (up to 70 years old) were similar to those of the Australian general population when adjusting for other factors (maintaining high CD4 cell count, low viral load, no prior ADI and non IDU-exposure). We found no evidence of increasing mortality compared to general population rates associated with age.

Some caution should be used when extrapolating such trends given the relatively limited history of HIV infection. To compare, type 1 childhood-onset diabetes patients have relatively low and stable mortality rates at earlier durations (up to 15 years) but increased rates at extended durations (up to 45 years) [23]. Concomitant illnesses are well documented in diabetic populations and SMRs for ischaemic heart disease and renal disease in particular, are much higher than those of the general population [24] and there are poorer prognoses compared to similar events in the general population [25]. In contrast, our findings are based on shorter duration of illness and it is possible that significant changes in morbidity and mortality in HIV–positive populations compared to the general population will emerge with extended follow-up.

The findings of this study apply generally to the AHOD population and are subject to the real life prospect that patients do not always adhere to therapy. In AHOD, while time off treatment is well reported, there are limited measures of the extent to which this is associated with adherence. However, the use of intent-to-continue treatment principles in this report reflects the applied utility of cART and results can be reasonably simply interpreted as describing the mortality risk in all patients who start cART.

Generally AHOD adherence is expected to be high for several reasons including low rates of loss-to-follow-up, cohort effect, and resourcing.

There are some limitations to our analyses. First, our data had insufficient observations to robustly assess survival at ages over 70. Some studies have accounted for insufficient data at these ages by applying assumed relative rates of mortality [26]. We believe there is high risk of error in doing so when comparing survival relative to the general population because at these ages, general mortality rates increase rapidly and can magnify errors in model assumptions. Further, the projection of whole of life experience to recent infections is likely to omit the effects of future developments in HIV specific and general treatments. Instead we estimated 10 year survival probabilities by age and relative to the general population. We believe this is a much more robust comparison as it does not require extensive extrapolation outside the range of the analysis dataset. This comparison found that estimated ten-year survival probabilities are close to population rates when controlling for other prognostic factors (age, mode of HIV exposure through IDU, ADI, HBVsAg, CD4 cell count, viral load and regimen number), and survival rates do not decrease relative to those of the general population at increased ages (at least up to 70 years of age). However, increased data on aged patients is required to more broadly describe survival experience in AHOD.

Second, the generalisation of these findings may be limited by likely survivor bias in our analysis associated especially with patients who commenced cART prior to 1999. AHOD commenced in 1999 nearly 5 years after the inception of cART and does not include mortality from before this time in patients (who were also receiving suboptimal treatment). However, a sensitivity analysis of patients who had commenced cART after 1999 was qualitatively similar to the model used in the primary analysis suggesting that the effects of survivor bias are limited. Also, the year of cART commencement and duration of treatment were not important as covariates in survival models for the study population especially when adjusting for recent CD4 cell count.

Third, the omission of the effects of prognostic lifestyle factors may reduce the accuracy of analyses, especially where the distribution of these factors differs from that of the general population. In particular, smoking is not widely recorded in AHOD. In a subsample of the cohort for which these rates were collected, 25% of patients had reported as a smoker at most recent visit, and 40% as ex-smokers, compared to 22% and 29% of males in the general Australian population 2007–2008 [27]. Had analyses been adjusted for smoking in this study, estimated survival probabilities could possibly exceed those seen in the general population when also controlling for other prognostic factors. Similarly this might be expected when adjusting for other lifestyle factors such as recent injecting drug use, alcohol consumption etc.

A strength of our analyses is the long-term follow-up compared to other studies, as few cohorts have data on patients who have received cART for 15+ years. Also, while randomised clinical trials have been used to investigate mortality among adults infected with HIV receiving cART [28], [29], they are generally limited to the examination of short-term effects. This analysis was able to examine survival in HIV-positive patients across long-term cART experience, and wide age ranges.

In conclusion, mortality rates in this large cohort of Australian HIV-positive individuals were close to those of the Australian population, particularly for patients with CD4 counts above 500 cells/µL. These rates did not vary much by duration of treatment. Changes in mortality with age were similar to those in the Australian general population. Further long-term follow-up is needed to characterize survival and other outcomes in HIV-positive patients on cART at older ages.
